# Diterpenoid from *Croton tonkinensis* as a Potential Radiation Sensitizer in Oral Squamous Cell Carcinoma: An In Vitro Study

**DOI:** 10.3390/ijms252111839

**Published:** 2024-11-04

**Authors:** Hui-Ming Lee, Ping-Chung Kuo, Wen-Hui Chen, Po-Jen Chen, Sio-Hong Lam, Yu-Chieh Su, Chih-Hao Chen

**Affiliations:** 1 Division of General Surgery, Department of Surgery, E-Da Cancer Hospital, I-Shou University, Kaohsiung 824005, Taiwan; ed106128@edah.org.tw; 2 School of Chinese Medicine for Post Baccalaureate, College of Medicine, I-Shou University, Kaohsiung 84001, Taiwan; 3 School of Pharmacy, College of Medicine, National Cheng Kung University, Tainan 701, Taiwan; z10502016@email.ncku.edu.tw (P.-C.K.); shlam@mail.ncku.edu.tw (S.-H.L.); 4 Department of Dentistry, E-Da Hospital, I-Shou University, Kaohsiung 824005, Taiwan; ed102129@edah.org.tw; 5 School of Medicine, College of Medicine, I-Shou University, Kaohsiung 82445, Taiwan; 6 Department of Medical Research, E-Da Hospital, Kaohsiung 824, Taiwan; ed113510@edah.org.tw; 7 Division of Hematology-Oncology, Department of Internal Medicine, E-Da Hospital, Kaohsiung 824410, Taiwan; 8 Department of Thoracic Surgery, Mackay Memorial Hospital, Taipei 104, Taiwan; 9 Department of Medicine, Mackay Medical College, New Taipei City 252, Taiwan

**Keywords:** oral squamous cell carcinoma (OSCC), natural sources, *Croton tonkinensis*, *Curcuma longa*, radiosensitization

## Abstract

Radiotherapy combined with a radiosensitizer represents an important treatment for head and neck squamous cell carcinoma (HNSCC). Only a few chemotherapy agents are currently approved as radiosensitizers for targeted therapy. Oral squamous cell carcinoma is one of the deadliest cancers, with approximately ~500,000 new diagnosed cases and 145,000 deaths worldwide per year. The incidence of new cases continues to increase in developing countries. This study aimed to investigate the effect of *Croton tonkinensis* and *Curcuma longa* on cell viability in OSCC cells. The HNSCC cell line OML1 and its radiation-resistant clone OML1-R were used. The anticancer effect and the mechanism of action of *Croton tonkinensis* and *Curcuma longa* in OSCC cells were analyzed by using cell viability assays, Western blot analysis, and Tranwell migration assays. The results showed that *Croton tonkinensis* concentration-dependently reduced the viability of OML1 and OML1-R (radioresistant) cells by downregulating the levels of AKT/mTOR mediators, such as p110α, p85, pAKT (ser473), p-mTOR (ser2448), and p-S6 Ribosomal (ser235/236). We found that cotreatment of OML1 and OML1R cells with either zVAD-FMK (apoptosis inhibitor), Ferrostatin-1 (Fer-1, a ferroptosis inhibitor), or chloroquine (CQ, an autophagy inhibitor) markedly reduced cell death. These results demonstrate that *Croton tonkinensis* exhibits anti-proliferation activity and highlight the therapeutic potential of small-molecule inhibitors against PI3K/mTOR signaling for radiosensitization in HNC treatment.

## 1. Introduction

Head and neck cancer is one of the globally significant public health concerns. According to research statistics from international public health in 2018, there are over 650,000 new cases of oral and pharyngeal cancer diagnosed worldwide annually, with approximately 50% of patients succumbing to the disease. The crude incidence rate is approximately 9 cases per 100,000 individuals globally. In Taiwan, according to the latest cancer registry reports from the Ministry of Health and Welfare in 2017, there were nearly 7800 cases of oral cancer, resulting in a crude incidence rate of approximately 33.08 cases per 100,000 individuals, significantly higher than the global incidence rate. Oral cancer ranks fifth among the top ten cancers in Taiwan, and the incidence rate among Taiwanese males is ten times higher than that of females, consistently ranking as the fourth most common cancer among Taiwanese males for twelve consecutive years [1].

Head and neck cancer can be classified into various types depending on the primary site, including oral cancer, nasopharyngeal cancer, oropharyngeal cancer, hypopharyngeal cancer, and laryngeal cancer. Over 90% of head and neck cancers are squamous cell carcinomas (HNSCCs). The major risk factors include betel nut chewing, smoking, alcohol consumption, and infection with human papillomavirus 16 (HPV16) [2]. Patients with early-stage disease are typically treated primarily with radiation or surgery. For locally advanced disease or cases with lymph node metastasis or distant metastasis, treatment often involves a combination of surgery, chemotherapy, and radiation therapy. However, the recurrence rate after treatment is relatively high, and the key determinant of local control in HNSCC depends on the tumor’s sensitivity to radiation [3]. Radioresistance has been a longstanding challenge in the clinical management of head and neck cancer. Tumor cells may proliferate again after receiving radiotherapy, especially in epithelial cell tumors such as head and neck, breast, and lymphoma, leading to local recurrence and treatment failure. Hence, there is a continual need to develop new drugs or treatment modalities. However, cancer therapy faces two main problems: the emergence of treatment-resistant cancer cells over time and nonspecific toxicity to normal cells [4]. Therefore, there is a growing interest in exploring natural compounds derived from plants to sensitize tumor cells to chemotherapy and/or radiotherapy [5]. Many plant-derived compounds, such as genistein from soybeans, curcumin from turmeric, resveratrol from grapes, and silymarin from milk thistle, have shown pharmacological safety profiles. They can sensitize tumor cells to chemotherapy and radiation therapy by inhibiting pathways that lead to treatment resistance [6]. The primary mechanism of radiation resistance in cancer cells is the activation of the phosphoinositide 3-kinase (PI3K)/thymoma viral proto-oncogene 1 (AKT)/ mammalian target of rapamycin (mTOR) pathway [7,8], and blocking this pathway has been observed to increase sensitivity to radiation and improve the effect of RT on tumor cells. In previous studies, we used mTOR-specific and dual-targeted PI3K/AKT inhibitors in oral cancer cell lines and found that blocking PI3K/mTOR increased the radiation sensitivity of oral cancer cells and reversed radiation resistance in a resistant strain (OML1-R) [9,10,11,12]. Notably, we identified the modulation of checkpoint kinase 2 (CHK2) activity and the resulting effects on cyclin-dependent protein kinase 1 (CDK1)/cyclin B1 activity as a potential mechanism [10,11], and we concluded that cell cycle arrest is the major mechanism of radiation sensitization associated with PI3K/mTOR inhibition. In the current study, we assessed the potential anti-tumor and radiation resistance-reversing effects of the CDK4/6 inhibitor LEE011, and explored the mechanism of cell cycle arrest in radioresistance in HNSCC. This study provides important clinical information regarding the development of radiosensitizer drugs for HNSCC therapy.

## 2. Results

### 2.1. NCKU_PCKuo _0001 and 0002 Induce Cytotoxicity in Human Squamous Carcinoma Cell

**Figure 1:**
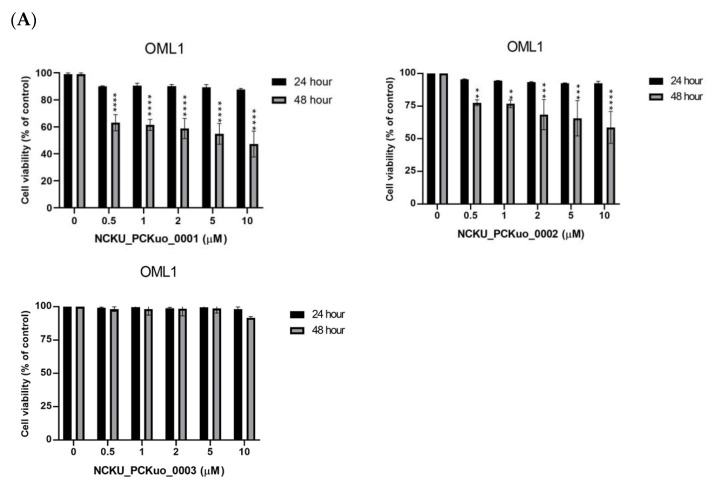

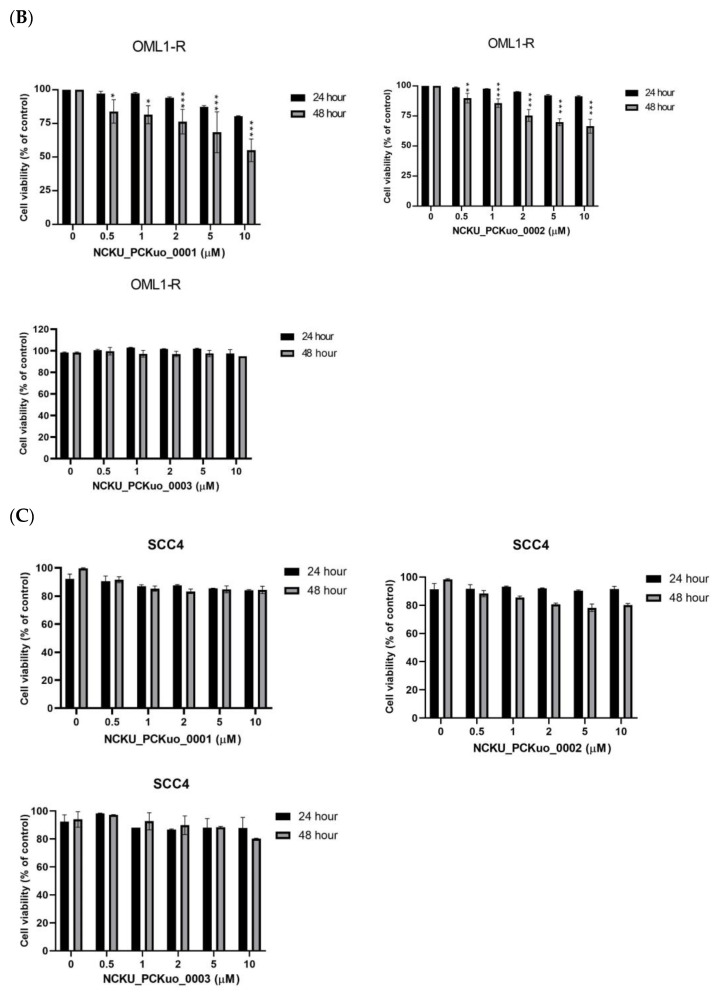

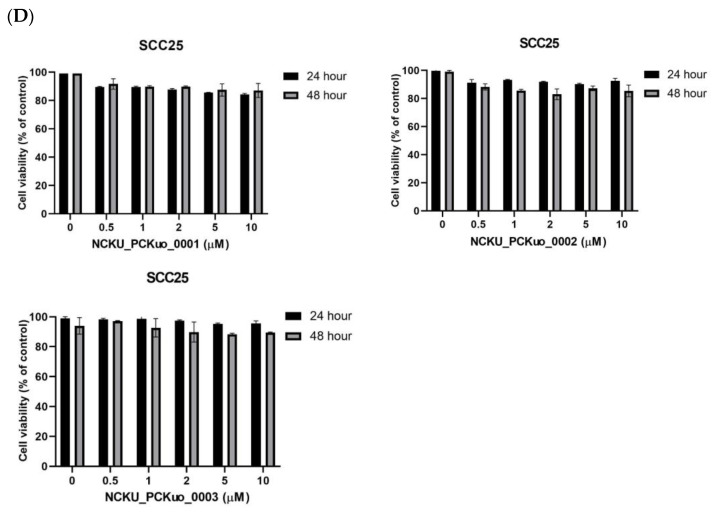
NCKU_PCKuo_0001, NCKU_PCKuo_0002, and NCKU_PCKuo_0003 induce cell cytotoxicity in oral squamous carcinoma cells. OML1 (**A**), OML-R (**B**), SCC4 (**C**), and SCC25 (**D**) cells were treated with the indicated doses of NCKU_PCKuo_0001 and NCKU_PCKuo_0002 for 24 and 48 h. Cell viability was examined by using the MTT assay. The values are deemed significantly different at * *p* < 0.05, ** *p* < 0.01, *** *p* < 0.001 and **** *p* < 0.0001.

In previous studies, the compounds ent-18-acetoxy-7α-hydroxykaur-16-en-15-one, ent-1β-acetoxy-7α,14β-dihydroxykaur-16-en-15-one, and curcumin displayed the most significant inhibition of superoxide anion generation and elastase release, though more toxic effects were also observed [13,14,15] (Table 1). To confirm the effects of NCKU_PCKuo_0001, NCKU_PCKuo_0002, and NCKU_PCKuo_0003 on the growth and survival of the parental OML1 cell line and the radioresistant phenotype of the OML1-R cell line, they were treated with 0, 0.5, 1, 2, 5, and 10 μM NCKU_PCKuo_0001 and NCKU_PCKuo_0002 for 24 and 48 h, respectively. As shown in Figure 1, NCKU_PCKuo_0001 and NCKU_PCKuo_0002 induced significant cytotoxicity in both oral carcinoma cell lines.

### 2.2. NCKU_PCKuo_0001- and NCKU_PCKuo_0002-Induced Cell Death in Oral Squamous Carcinoma Cells

**Figure 2:**
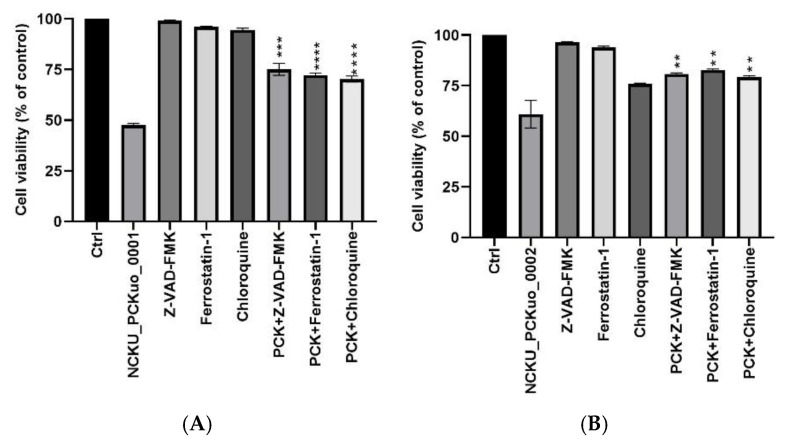

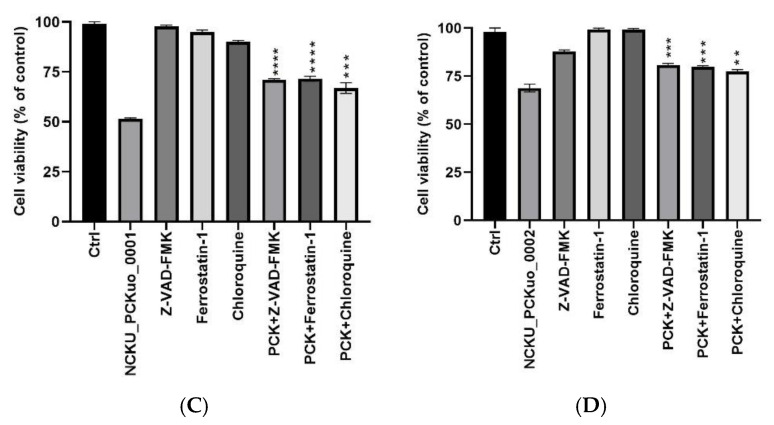
NCKU_PCKuo_0001, NCKU_PCKuo_0002, and NCKU_PCKuo_0003 induce cell cytotoxicity in oral squamous carcinoma cells. OML1 (**A**,**B**) and OML-R (**C**,**D**) cells were treated with the indicated doses of NCKU_PCKuo_0001 and NCKU_PCKuo_0002 for 24 and 48 h. Cell viability was examined by using the CCK-8 assay. The values are deemed significantly different at ** *p* < 0.01, *** *p* < 0.001 and **** *p* < 0.0001.

To identify whether NCKU_PCKuo_0001- and NCKU_PCKuo_0002-induced cell death is regulated by a pan-caspase inhibitor (Z-VAD-FMK) or chloroquine (CQ, an autophagy inhibitor) and Ferrostatin-1 (Fer-1, a ferroptosis inhibitor), we treated the OSCC cells with NCKU_PCKuo_0001 and NCKU_PCKuo_0002 (10 μM, 48 h) and pre-treated them with Z-VAD-FMK (20 μM), CQ (10 μM), and Fer-1 (10 μM) before NCKU_PCKuo treatment (Figure 2). This result indicates that Z-VAD-FMK, CQ, and Fer-1 inhibit the decrease in cell viability.

### 2.3. Inhibition of the AKT/mTOR Signaling Pathway Sensitizes Radioresistant Cells to IR

**Figure 3:**
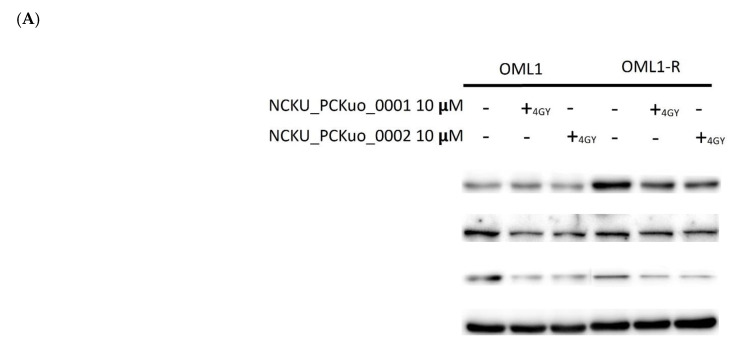

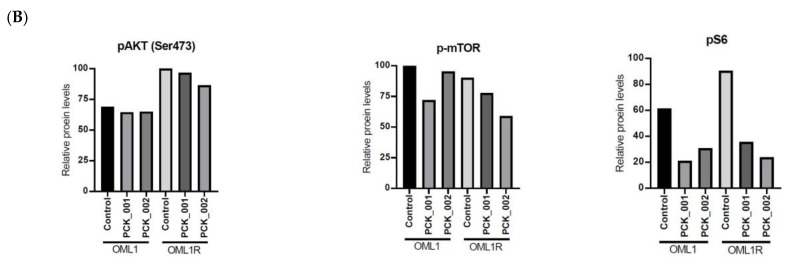
NCKU_PCKuo_0001 and NCKU_PCKuo_0002 inhibited pAKT(Ser473), p-mTOR and ps6 signaling in OML1-R cells. (**A**) RT-resistant OML1-R cells were treated with 10 μM of NCKU_PCKuo_0001 and NCKU_PCKuo_0002 and without 4 Gy RT by western blot. (**B**) The levels of pAKT(Ser473), p-mTOR and pS6 were analyzed by western blotting with specific antibodies.

To confirm the radioresistant phenotype of the OML1-R cell line, we determined the plating efficiency of the parental OML1 cells and the radioresistant OML-1R subline cells that were cultured after high-dose fractionated IR exposure (4 Gy) and then examined them using the clonogenic survival assay. Next, we determined that NCKU_PCKuo_0001 and NCKU_PCKuo_0002 at 10 μM in combination with IR dramatically inhibited the proliferation of OML1 and OML1-R cells. We also analyzed the expression profiles of AKT/mTOR signaling pathway-related proteins; pAKT (Ser 473) and p-S6 showed decreased expression levels in OML1-R cells (Figure 3). Thus, our findings suggest that the PI3K/AKT/mTOR signaling pathway is actively involved in OSCC radioresistance.

### 2.4. NCKU_PCKuo_0001 and NCKU_PCKuo_0002 Reduced Cell Migration and Invasion

**Figure 4:**
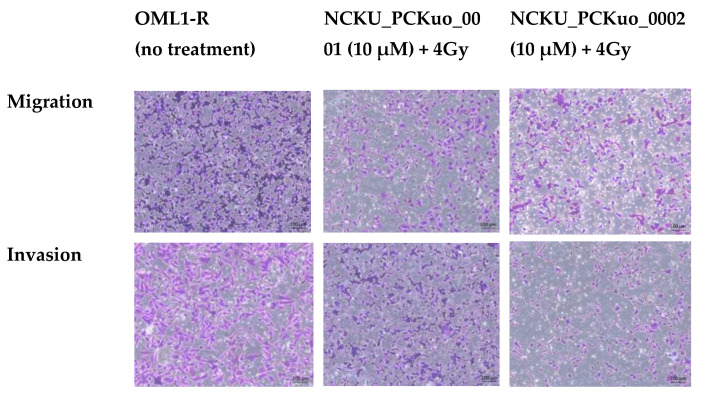
Migration and invasion abilities of OML1-R cells according to NCKU_PCKuo_0001 and NCKU_PCKuo_0002 treatment. RT-resistant OML1-R cells were treated with 10 μM of NCKU_PCKuo_0001 and NCKU_PCKuo_0002 and without 4 Gy RT.

Radiation-enhanced cell metastasis and invasion is one important reason for the poor prognosis of tumors. Transwell migration assays were used to evaluate the effect of NCKU_PCKuo_0001 and NCKU_PCKuo_0002 on migration and invasion in OML1-R cells. Additionally, a Transwell migration assay performed to determine if NCKU_PCKuos had an effect on the metastatic properties of OSCC cells showed that the number of migrated cells decreased significantly in NCKU_PCKuo_0001- and NCKU_PCKuo_0002-treated OML1-R cells relative to the number in the control cells. Matrigel was coated on the upper chamber to stimulate the basement membrane in invasion assays. The results revealed that NCKU_PCKuo_0001 and NCKU_PCKuo_0002 decreased the number of cells invading the Matrigel-coated filters. Moreover, the number of invaded cells decreased significantly in OML1-R cells treated with NCKU_PCKuo_0001 and NCKU_PCKuo_0002 (Figure 4). These findings demonstrate that NCKU_PCKuo_0001 and NCKU_PCKuo_0002 play a role in the metastatic properties of OSCC.

## 3. Discussion

Radiotherapy and surgery are both commonly used treatments for patients with head and neck cancer, with outcomes depending significantly on the resources and expertise available at the treating facility. During surgery, the cancerous tissue in the local area, along with the affected lymph nodes, is removed. This surgical approach is frequently paired with adjuvant or neoadjuvant radiotherapy, chemotherapy, or a combination of both [16]. Patients with head and neck squamous cell carcinoma (HNSCC) who undergo radiation therapy or chemotherapy often suffer from severe side effects, such as mucositis, dysphagia, leukopenia, and thrombocytopenia [17]. These side effects elevate the risk of infection and bleeding, consequently diminishing the patients’ health-related quality of life. Several signaling pathways, including those involved in apoptosis, metastasis, DNA repair, and protein degradation, have been identified as key factors influencing the effectiveness of radiotherapy. Recent research into the mechanisms of radioresistance has highlighted the role of the PI3K/AKT/mTOR pathways in determining radiosensitivity [18,19]. Our previous studies identified RAD001 [20], AZD2014 [21], and BEZ235 [22] as promising small-molecule drugs for enhancing radiosensitivity. Here, we report that *Croton tonkinensis* promotes radiation sensitization through a common mechanism: the AKT/mTOR pathway. Using OML1-R and OML1 cell lines, we developed a model to evaluate the radiosensitizing effects of various drugs in head and neck squamous cell carcinoma (HNSCC) [9,10,11,23]. Our prior research explored the radiosensitizing impact of PI3K/mTOR/CDK inhibition, identifying cell cycle arrest as the key mechanism. CDK4/6 inhibitors emerged as the most effective and least toxic targets, though their clinical application requires further investigation through clinical trials. Currently, chemotherapeutic agents such as cetuximab, cisplatin, and fluorouracil are the most commonly approved drugs for use in concurrent chemoradiotherapy for head and neck cancer [24]. These drugs have been thoroughly studied for their safety and pharmacokinetics. However, while integrating them into clinical practice may be straightforward, higher toxicity levels have been reported in patients receiving concurrent chemoradiotherapy. Additionally, studies on other small-molecule agents, such as hypoxic radiosensitizers [25] like tirapazamine [26] and nimorazole [27], have not demonstrated a significant overall survival benefit. Our research indicates the potential of using NCKU_PCKuo_0001 and 0002 in concurrent radiotherapy, offering a novel treatment approach for advanced or recurrent oral squamous cell carcinoma.

## 4. Materials and Methods

### 4.1. Reagents and Chemicals

NCKU_PCKuo_0001 (ent-18-acetoxy-7α-hydroxykaur-16-en-15-one), NCKU_PCKuo_0002 (ent-1β-acetoxy-7α,14β-dihydroxykaur-16-en-15-one), and NCKU_PCKuo_0003 (curcumin) were provided by the National Cheng Kung University Clinical Pharmacy and Pharmaceutical Sciences [13]. Stock solutions were prepared in dimethylsulfoxide (DMSO) at 10 mM, stored at −20 **°C** until further use, and diluted in culture medium for each experiment.

### 4.2. Cell Lines and Culture

The head and neck squamous cell carcinomas (HNSCCs) SCC4 (CRL-1624) and SCC25 (CRL-1628) derived from a squamous cell carcinoma of the tongue were purchased from the ATCC. The cells were cultured in DMEM/F12 containing 10% FBS, 1% penicillin–streptomycin, and 2 mM L-glutamine. OML 1 and OML 1-R cells were established as previously described and maintained in RPMI1640 containing 10% FBS, 1% penicillin–streptomycin, and 2 mM L-glutamine. The cells were cultured in a humidified atmosphere of 5% CO_2_ at 37 °C.

### 4.3. Cell Counting Kit-8 (CCK-8) Assay

The CCK-8 assay (Thermo Fisher Scientific, Inc., Waltham, MA, USA, catalogue PA5-32348) was performed to detect cell viability. Briefly, 2 × 10^4^ cells were seeded in a 96-well plate for overnight culture, and then treated with either DMSO or NCKU_PCK for 24 and 48 h. Cell viability was measured after treatment by adding 10 μL of CCK-8 solution to the culture medium. After incubation for 3 h at 37 °C, the absorbance was measured by using a microplate ELISA reader at a wavelength of 450 nm with a reference wavelength of 650 nm.

### 4.4. Transwell Migration Assays

Cell migratory ability was evaluated with the use of the Transwell polycarbonate membrane inserts (Millipore, Billerica, MA, USA). The cells were planted into the upper chamber of the insert. Then, 10% FBS was used to supplement cell-free medium in the low chamber at 37 °C for 1 day. Cells migrating through the membranes were cultured in 4% paraformaldehyde and stained with 0.1% crystal violet. For the cell invasion assay, 1 × 105 cells in sterile medium were seeded into the upper chamber with Martigel (Corning^®^, Corning, NY, USA). Both migrated and invaded cells were observed and counted under an inverted light microscope at a magnification of ×200.

### 4.5. Western Blotting

Cells were harvested and lysed with cell lysis buffer (50 mM Tris-HCl pH 7.4, 150 mM Nacl, 2 mM EDTA pH 8, 0.5% Nonidet P-40, and 1% Trion-X 100) after treatment. Protein concentration were measured using the DC protein assay (Bio-Rad, Richmond, CA, USA). A total of 20 μg of protein from each sample was separated by 10% sodium dodecylsulphate-polyacrylamide gel electrophoresis and transferred to a polyvinylidene difluoride membrane (Millipore, Billerica, MA, USA). Membranes were incubated with the corresponding primary antibodies and HRP-conjugated secondary antibodies after blocking with non-fat dry milk for 1 h. The following antibodies were used: anti-PI3 Kinase p110α (catalogue #4249), anti-PI3 kinase p85 (19H8) (catalogue #4257), anti-AKT1 (phosphor S473) (catalogue #4260), anti-phospho-mTOR (Ser2448) (catalogue #2971), anti-phospho S6 (Ser235/236) (catalogue #2211) (all at 1:1000; Cell Signaling Tech, Beverly, MA, USA), and anti-GAPDH (1:10,000; Elabscience^®^, Houston, TX, USA) (E-AB-48017). Proteins were visualized using a chemiluminescence (ECL) (T-Pro Biotechnology, Xinbei, Taiwan, No JT-96-K004M) detection kit and all blots were quantified with ImageJ bundled with 64-bit Java8 (National Institutes of Health, Bethesda, MD, USA).

### 4.6. Statistical Analysis

All data are presented as the mean ± standard deviation. Significance levels were calculated using Student’s *t*-test and *p*-values of less than 0.05 were considered statistically significant.

## 5. Conclusions

The present results focus on the inhibition of oral squamous carcinoma cells (OML1) and the radiosensitization of OML1-R cells by NCKU_PCK_0001 and NCKU_PCK_0002. These compounds reduced cell viability, migration, and invasion by downregulating the AKT/mTOR signaling pathway. The findings suggest that NCKU_PCK_0001 and NCKU_PCK_0002 enhance the anticancer effects on oral squamous carcinoma cells. Validation of these results using an animal model should be considered in future studies.

## Figures and Tables

**Table 1:** Chemical structures of NCKU_PCKuo_0001, NCKU_PCKuo_0002, and NCKU_PCKuo_0003 from ent-18-acetoxy-7α-hydroxykaur-16-en-15-one, ent-1β-acetoxy-7α,14β-dihydroxykaur-16-en-15-one, and curcumin.

No.	Structure	Name
NCKU_PCKuo_0001	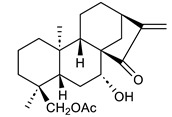	ent-18-acetoxy-7α-hydroxykaur-16-en-15-one
NCKU_PCKuo_0002	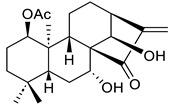	ent-1β-acetoxy-7α,14β-dihydroxykaur-16-en-15-one
NCKU_PCKuo_0003	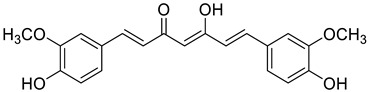	curcumin

## Data Availability

Data is contained within the article.

## References

[1] Gormley M., Creaney G., Schache A., Ingarfield K., Conway D.I. (2022). Reviewing the epidemiology of head and neck cancer: Definitions, trends and risk factors. Br. Dent. J..

[2] Johnson D.E., Burtness B., Leemans C.R., Lui V.W.Y., Bauman J.E., Grandis J.R. (2020). Head and neck squamous cell carcinoma. Nat. Rev. Dis. Primers.

[3] Hutchinson M.N.D., Mierzwa M., D’Silva N.J. (2020). Radiation resistance in head and neck squamous cell carcinoma: Dire need for an appropriate sensitizer. Oncogene.

[4] Talib W.H., Alsayed A.R., Barakat M., Abu-Taha M.I., Mahmod A.I. (2021). Targeting Drug Chemo-Resistance in Cancer Using Natural Products. Biomedicines.

[5] Imran M., Insaf A., Hasan N., Sugandhi V.V., Shrestha D., Paudel K.R., Jha S.K., Hansbro P.M., Dua K., Devkota H.P. (2023). Exploring the Remarkable Chemotherapeutic Potential of Polyphenolic Antioxidants in Battling Various Forms of Cancer. Molecules.

[6] Wang Z., Dabrosin C., Yin X., Fuster M.M., Arreola A., Rathmell W.K., Generali D., Nagaraju G.P., El-Rayes B., Ribatti D. (2015). Broad targeting of angiogenesis for cancer prevention and therapy. Semin. Cancer Biol..

[7] Willems L., Tamburini J., Chapuis N., Lacombe C., Mayeux P., Bouscary D. (2012). PI3K and mTOR signaling pathways in cancer: New data on targeted therapies. Curr. Oncol. Rep..

[8] Ahmad A., Biersack B., Li Y., Kong D., Bao B., Schobert R., Padhye S.B., Sarkar F.H. (2013). Deregulation of PI3K/Akt/mTOR signaling pathways by isoflavones and its implication in cancer treatment. Anticancer. Agents Med. Chem..

[9] Yu C.C., Hung S.K., Lin H.Y., Chiou W.Y., Lee M.S., Liao H.F., Huang H.B., Ho H.C., Su Y.C. (2017). Targeting the PI3K/AKT/mTOR signaling pathway as an effectively radiosensitizing strategy for treating human oral squamous cell carcinoma in vitro and in vivo. Oncotarget.

[10] Yu C.C., Huang H.B., Hung S.K., Liao H.F., Lee C.C., Lin H.Y., Li S.C., Ho H.C., Hung C.L., Su Y.C. (2016). AZD2014 Radiosensitizes Oral Squamous Cell Carcinoma by Inhibiting AKT/mTOR Axis and Inducing G1/G2/M Cell Cycle Arrest. PLoS ONE.

[11] Yu C.C., Hung S.K., Liao H.F., Lee C.C., Lin H.Y., Lai H.C., Li S.C., Ho H.C., Huang H.B., Su Y.C. (2014). RAD001 enhances the radiosensitivity of SCC4 oral cancer cells by inducing cell cycle arrest at the G2/M checkpoint. Anticancer Res..

[12] American Association for Cancer Research (2016). Targeting Mutant AKT in Cancer. Cancer Discov..

[13] Kuo P.C., Yang M.L., Hwang T.L., Lai Y.Y., Li Y.C., Thang T.D., Wu T.S. (2013). Anti-inflammatory diterpenoids from *Croton tonkinensis*. J. Nat. Prod..

[14] Minh P.T., Ngoc P.H., Taylor W.C., Cuong N.M. (2004). A new ent-kaurane diterpenoid from *Croton tonkinensis* leaves. Fitoterapia.

[15] Giang P.M., Jin H.Z., Son P.T., Lee J.H., Hong Y.S., Lee J.J. (2003). ent-Kaurane diterpenoids from croton tonkinensis inhibit LPS-induced NF-kappaB activation and NO production. J. Nat. Prod..

[16] Lavaf A., Genden E.M., Cesaretti J.A., Packer S., Kao J. (2008). Adjuvant radiotherapy improves overall survival for patients with lymph node-positive head and neck squamous cell carcinoma. Cancer.

[17] Geiger J.L., Bauman J.E., Gibson M.K., Gooding W.E., Varadarajan P., Kotsakis A., Martin D., Gutkind J.S., Hedberg M.L., Grandis J.R. (2016). Phase II trial of everolimus in patients with previously treated recurrent or metastatic head and neck squamous cell carcinoma. Head Neck.

[18] Fritsch C., Huang A., Chatenay-Rivauday C., Schnell C., Reddy A., Liu M., Kauffmann A., Guthy D., Erdmann D., De Pover A. (2014). Characterization of the novel and specific PI3Kα inhibitor NVP-BYL719 and development of the patient stratification strategy for clinical trials. Mol. Cancer Ther..

[19] Burris H.A. (2013). Overcoming acquired resistance to anticancer therapy: Focus on the PI3K/AKT/mTOR pathway. Cancer Chemother. Pharmacol..

[20] Freudlsperger C., Burnett J.R., Friedman J.A., Kannabiran V.R., Chen Z., Van Waes C. (2011). EGFR-PI3K-AKT-mTOR signaling in head and neck squamous cell carcinomas: Attractive targets for molecular-oriented therapy. Expert. Opin. Ther. Targets.

[21] Khan K.H., Yap T.A., Yan L., Cunningham D. (2013). Targeting the PI3K-AKT-mTOR signaling network in cancer. Chin. J. Cancer.

[22] Skvortsov S., Dudás J., Eichberger P., Witsch-Baumgartner M., Loeffler-Ragg J., Pritz C., Schartinger V.H., Maier H., Hall J., Debbage P. (2014). Rac1 as a potential therapeutic target for chemo-radioresistant head and neck squamous cell carcinomas (HNSCC). Br. J. Cancer.

[23] Lin H.Y., Hung S.K., Lee M.S., Chiou W.Y., Huang T.T., Tseng C.E., Shih L.Y., Lin R.I., Lin J.M., Lai Y.H. (2015). DNA methylome analysis identifies epigenetic silencing of FHIT as a determining factor for radiosensitivity in oral cancer: An outcome-predicting and treatment-implicating study. Oncotarget.

[24] Winquist E., Agbassi C., Meyers B.M., Yoo J., Chan K.K.W. (2017). Systemic therapy in the curative treatment of head and neck squamous cell cancer: A systematic review. J. Otolaryngol. Head Neck Surg..

[25] Zackrisson B., Nilsson P., Kjellén E., Johansson K.A., Modig H., Brun E., Nyman J., Friesland S., Reizenstein J., Sjödin H. (2011). Two-year results from a Swedish study on conventional versus accelerated radiotherapy in head and neck squamous cell carcinoma--the ARTSCAN study. Radiother. Oncol..

[26] Rischin D., Peters L.J., O’Sullivan B., Giralt J., Fisher R., Yuen K., Trotti A., Bernier J., Bourhis J., Ringash J. (2010). Tirapazamine, cisplatin, and radiation versus cisplatin and radiation for advanced squamous cell carcinoma of the head and neck (TROG 02.02, HeadSTART): A phase III trial of the Trans-Tasman Radiation Oncology Group. J. Clin. Oncol..

[27] Hassan Metwally M.A., Jansen J.A., Overgaard J. (2015). Study of the population pharmacokinetic characteristics of nimorazole in head and neck cancer patients treated in the DAHANCA-5 trial. Clin. Oncol. (R. Coll. Radiol.).

